# Supplementary material for: Mixed IgG Fc immune complexes exhibit blended binding profiles and refine FcR affinity estimates
Source: bioRxiv. 2023 Feb 15:2023.02.15.528730. Preprint. [Version 1] doi: 10.1101/2023.02.15.528730 (PMC9949097; doi:10.1101/2023.02.15.528730)
Supplement: Supplement 1 [file media-1.pdf]

Supplemental Figures

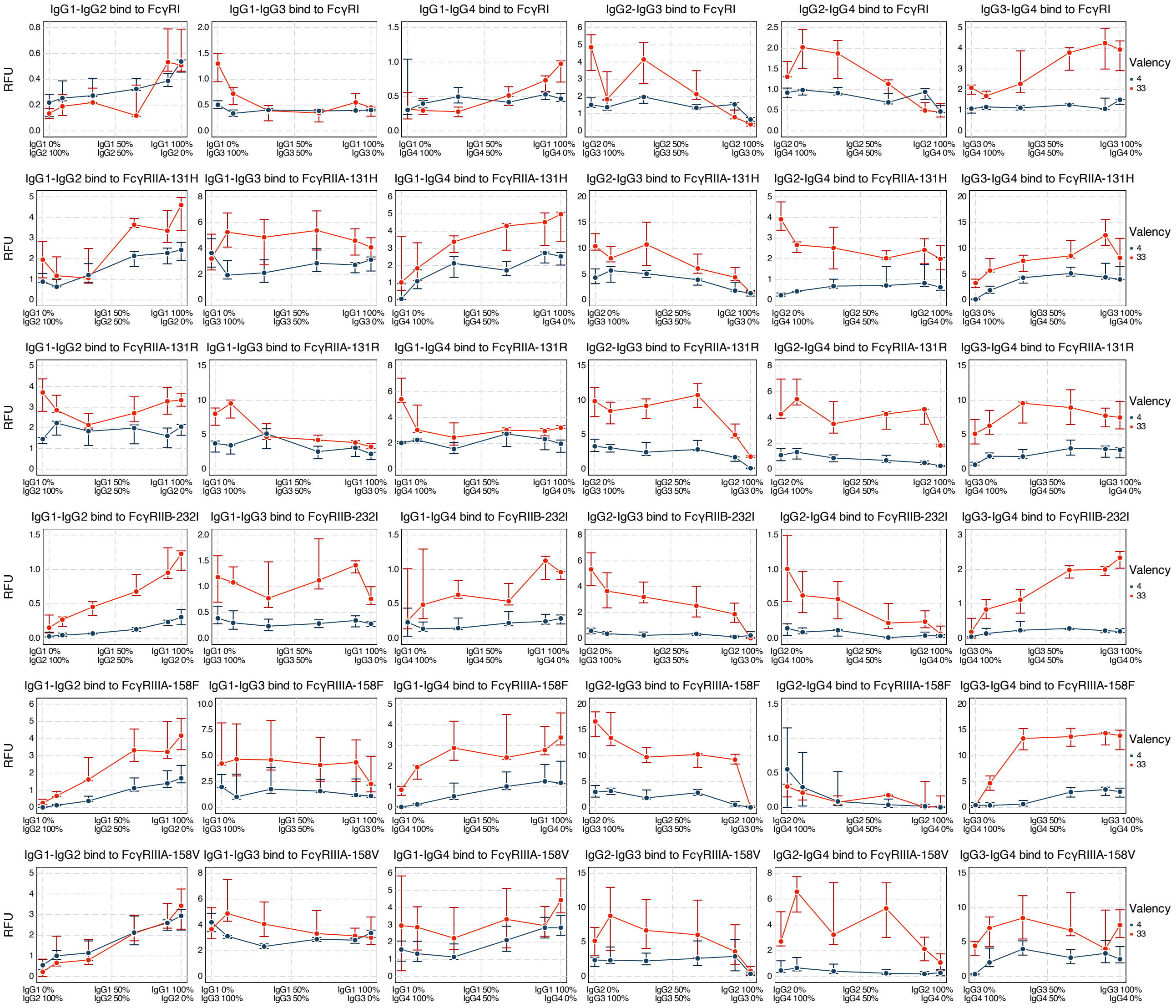

Figure S1: Experimental IC mixture binding data. Quantification of human IgG subclass pairs TNP-4-BSA and TNP-33-BSA IC binding to CHO cells expressing the indicated hFcγRs. Relative fluorescent units (RFU) of different multivalent immune complexes consisting of various IgG mixtures binding to different human immune cell receptors. Error bars indicate the biological replicates from experiments. Fluorescent values were normalized so that the daily geometric average measurements are 1.

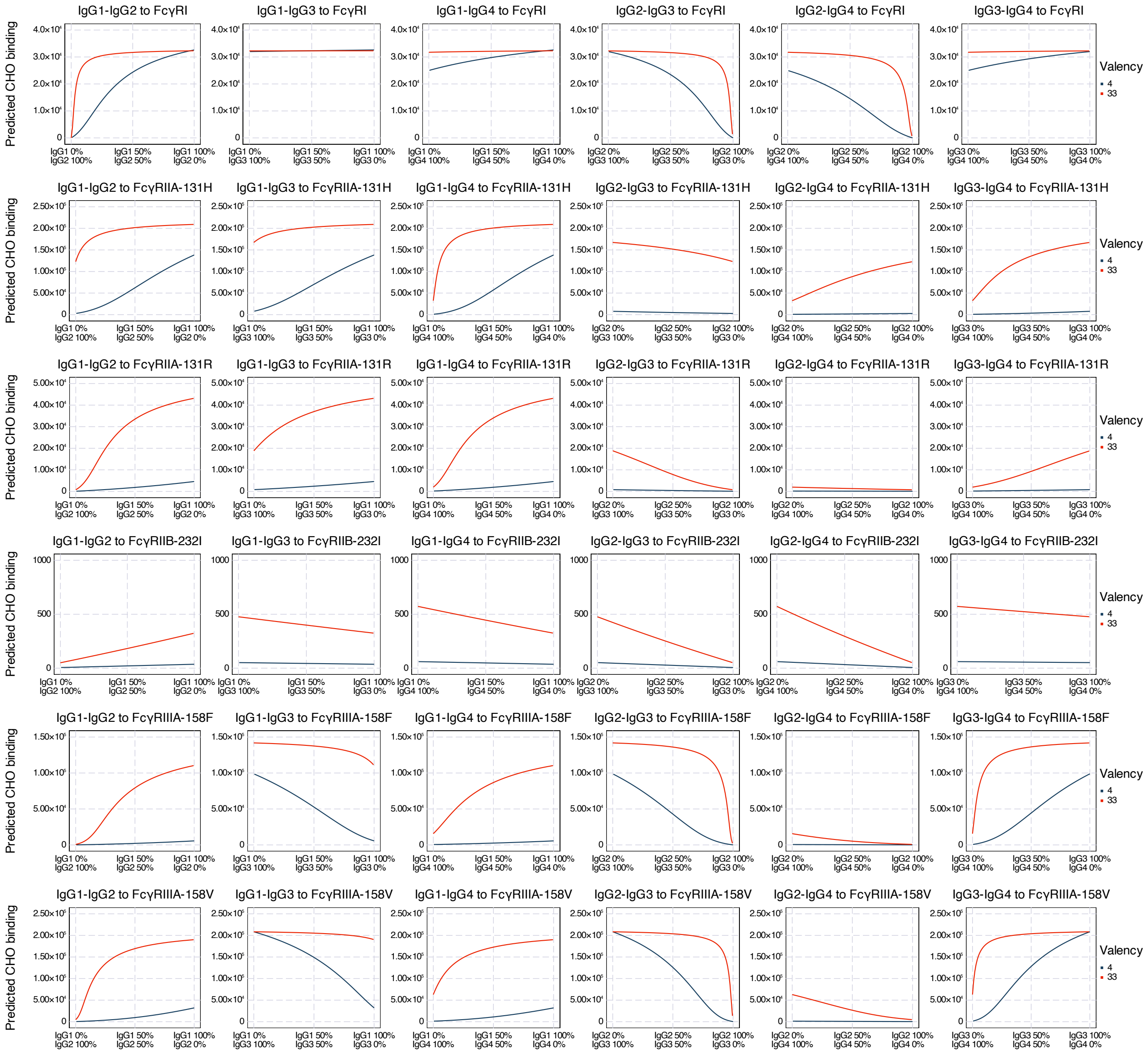

Figure S2: Predicted binding of IgG subclass mixtures with documented affinities. Amount of binding for complexes of each IgG subclass pairs binding to each CHO cell predicted by the multivalent binding model with the documented affinities. The receptor abundances were as geometric means of measurement.

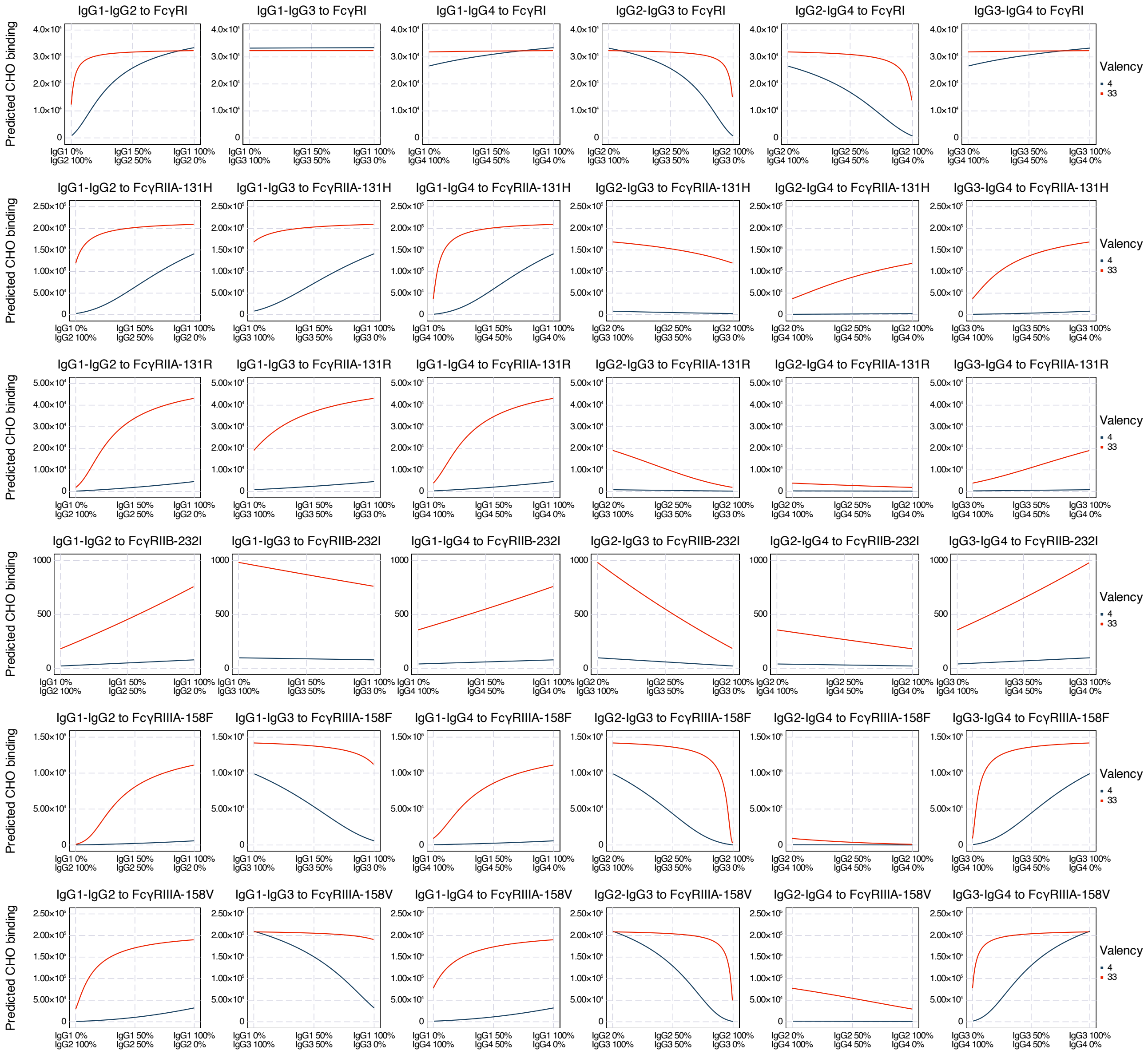

Figure S3: Predicted binding of IgG subclass mixtures with updated affinities. Amount of binding for complexes of each IgG subclass pairs binding to each CHO cell predicted by the multivalent binding model with the updated affinities. The receptor abundances were as geometric means of measurement.

Supplemental Tables

Table S1. One-way ANOVA performed on the measurements indicates that the majority of variance in measurements comes from between conditions.

Source	DF	SS	MSS	F	p
Condition	431	11110.23	25.778	6.00915	5.9926×10^{-125}
Residuals	1066	4572.88	4.290		
Total	1497	15683.11			

$R^2 = 0.7084$

Table S2. Geometric mean and inferred prior distribution of FcR abundance. Antibody binding capacity on CHO cells from measurements used in IgG mixture *in vitro* binding experiment. The geometric means were calculated from primary data published in previous work[1]. logN represents a lognormal distribution.

Receptor	Geometric mean	Inferred distribution
FcγRI	101494	logN(μ=11.53, σ=0.26)
FcγRIIA-131H	1006300	logN(μ=13.82, σ=0.27)
FcγRIIA-131R	190433	logN(μ=12.16, σ=1.46)
FcγRIIB-232I	75085	logN(μ=11.23, σ=1.40)
FcγRIIIA-158F	634324	logN(μ=13.36, σ=0.70)
FcγRIIIA-158V	979452	logN(μ=13.80, σ=0.38)

Table S3. Updated Fc affinities' interquartile ranges from their posterior distributions.

K_a (M^{-1})	IgG1	IgG2	IgG3	IgG4
FcγRI	$5.809 \sim 8.635 \times 10^7$	$1.189 \sim 1.911 \times 10^6$	$5.525 \sim 8.730 \times 10^7$	$3.012 \sim 4.770 \times 10^7$
FcγRIIA-131H	$4.780 \sim 5.929 \times 10^6$	$3.930 \sim 4.724 \times 10^5$	$8.622 \sim 9.621 \times 10^5$	$1.625 \sim 2.057 \times 10^5$
FcγRIIA-131R	$3.277 \sim 3.758 \times 10^6$	$1.719 \sim 2.339 \times 10^5$	$8.521 \sim 9.861 \times 10^5$	$2.833 \sim 3.864 \times 10^5$
FcγRIIB-232I	$2.166 \sim 3.053 \times 10^5$	$5.931 \sim 8.134 \times 10^4$	$2.714 \sim 3.702 \times 10^5$	$1.131 \sim 1.504 \times 10^5$
FcγRIIIA-158F	$1.106 \sim 1.288 \times 10^6$	$2.938 \sim 4.405 \times 10^4$	$7.243 \sim 8.329 \times 10^6$	$1.337 \sim 1.764 \times 10^5$
FcγRIIIA-158V	$1.913 \sim 2.107 \times 10^6$	$1.433 \sim 1.966 \times 10^5$	$0.919 \sim 1.073 \times 10^7$	$2.509 \sim 3.348 \times 10^5$

Table S4. Updated Fc affinities median values.

K_a (M^{-1})	IgG1	IgG2	IgG3	IgG4
FcγRI	7.140×10^7	1.496×10^6	6.998×10^7	3.835×10^7
FcγRIIA-131H	5.314×10^6	4.306×10^5	9.128×10^5	1.808×10^5
FcγRIIA-131R	3.503×10^6	2.005×10^5	9.178×10^5	3.291×10^5
FcγRIIB-232I	2.549×10^5	6.911×10^4	3.157×10^5	1.305×10^5
FcγRIIIA-158F	1.195×10^6	3.639×10^4	7.741×10^6	1.536×10^5
FcγRIIIA-158V	2.006×10^6	1.678×10^5	9.941×10^6	2.906×10^5

Table S5. Geometric mean and inferred prior distribution of FcR abundance. Antibody binding capacity on CHO cells used in validation with Robinett et al.[2] binding dataset. The geometric means were calculated from primary data published in previous work[1]. logN represents a lognormal distribution.

Receptor	Geometric mean	Inferred distribution
FcγRI	232872	logN(μ=12.36, σ=0.25)
FcγRIIA-131H	318819	logN(μ=12.67, σ=0.26)
FcγRIIA-131R	1605372	logN(μ=14.29, σ=0.14)
FcγRIIB-232I	394556	logN(μ=12.89, σ=0.45)
FcγRIIIA-158F	4677645	logN(μ=15.36, σ=0.22)
FcγRIIIA-158V	3680708	logN(μ=15.12, σ=0.25)

Table S6. Geometric means of measured FcγR expression, i.e. the number of quantified binding sites for the respective anti-FcR antibodies on effector cells calculated from the primary data published in previous work[1].

Receptor	Non-classical monocyte	Classical monocyte	Neutrophil
FcγRI	6326	84559	1847
FcγRIIA	82542	96646	158228
FcγRIIB	7140	5167	2351
FcγRIIIA	200213	19533	0*
FcγRIIIB	0*	0*	1299166

* Although one cannot have a geometric mean as 0, these values were consistently measured as non-expressed, so we used 0 as the value.

References

1. Kerntke, C., Nimmerjahn, F. & Biburger, M. There Is (Scientific) Strength in Numbers: A Comprehensive Quantitation of Fc Gamma Receptor Numbers on Human and Murine Peripheral Blood Leukocytes. *Front. Immunol.* **11**, (2020).
2. Robinett, R. A. *et al.* Dissecting FcγR Regulation through a Multivalent Binding Model. *Cell Syst.* **7**, 41-48.e5 (2018).
